# Epigenetic and Transcriptional Shifts in Human Neural Stem Cells after Reprogramming into Induced Pluripotent Stem Cells and Subsequent Redifferentiation

**DOI:** 10.3390/ijms25063214

**Published:** 2024-03-12

**Authors:** Carolin Haubenreich, Michael Lenz, Andreas Schuppert, Michael Peitz, Philipp Koch, Martin Zenke, Oliver Brüstle

**Affiliations:** 1 Institute of Reconstructive Neurobiology, University of Bonn Medical Faculty and University Hospital Bonn, 53127 Bonn, Germany; carolin.haubenreich@googlemail.com (C.H.); peitz@uni-bonn.de (M.P.); philipp.koch@zi-mannheim.de (P.K.); 2 Institute for Computational Biomedicine, RWTH Aachen University, 52074 Aachen, Germany; michael.l.lenz@googlemail.com (M.L.); schuppert@combine.rwth-aachen.de (A.S.); 3 Cell Programming Core Facility, University of Bonn Medical Faculty, 53127 Bonn, Germany; 4 Department of Cell Biology, Institute for Biomedical Engineering, RWTH Aachen University Medical School, 52074 Aachen, Germany; martin.zenke@rwth-aachen.de; 5 Helmholtz Institute for Biomedical Engineering, RWTH Aachen University, 52074 Aachen, Germany; 6 Department of Hematology, Oncology and Stem Cell Transplantation, RWTH Aachen University Medical School, 52074 Aachen, Germany; 7 Center for Integrated Oncology Aachen Bonn Cologne Düsseldorf (CIO ABCD), 52074 Aachen, Germany

**Keywords:** neural stem cells, reprogramming, iPS cells, pluripotency, X chromosome

## Abstract

Induced pluripotent stem cells (iPSCs) and their derivatives have been described to display epigenetic memory of their founder cells, as well as de novo reprogramming-associated alterations. In order to selectively explore changes due to the reprogramming process and not to heterologous somatic memory, we devised a circular reprogramming approach where somatic stem cells are used to generate iPSCs, which are subsequently re-differentiated into their original fate. As somatic founder cells, we employed human embryonic stem cell-derived neural stem cells (NSCs) and compared them to iPSC-derived NSCs derived thereof. Global transcription profiling of this isogenic circular system revealed remarkably similar transcriptomes of both NSC populations, with the exception of 36 transcripts. Amongst these we detected a disproportionately large fraction of X chromosomal genes, all of which were upregulated in iPSC-NSCs. Concurrently, we detected differential methylation of X chromosomal sites spatially coinciding with regions harboring differentially expressed genes. While our data point to a pronounced overall reinstallation of autosomal transcriptomic and methylation signatures when a defined somatic lineage is propagated through pluripotency, they also indicate that X chromosomal genes may partially escape this reinstallation process. Considering the broad application of iPSCs in disease modeling and regenerative approaches, such reprogramming-associated alterations in X chromosomal gene expression and DNA methylation deserve particular attention.

## 1. Introduction

Human induced pluripotent stem cells (iPSCs) provide a continuous donor source for the generation of specific somatic cell types for disease modeling and regeneration. Considering the harsh reprogramming procedure and the requirement of epigenetic remodeling for proper reprogramming, a critical question to be addressed is whether and to what extent alterations caused by reprogramming can confound readout parameters in disease modeling or influence the clinical safety of human iPSC-derived cell populations. Several studies of mouse and human systems addressed the equivalence of embryonic stem cells (ESCs) and iPSCs. Although iPSCs and ESCs are very similar concerning morphology, expression of pluripotency genes, and the ability to differentiate into all three germ layers, more detailed analyses of their differentiation potential, their transcriptomic and epigenetic signatures led to controversial results [1,2,3,4,5,6,7,8,9,10,11,12]. Global transcriptome analyses of several iPSC and ESC lines revealed deviations in hundreds to thousands of genes [1,2]. Remarkably, differentially expressed genes were also observed in ESCs and isogenic iPSCs generated from ESC-derived fibroblast-like cells [13,14]. However, these studies focused on the comparison of iPSCs with ESCs, an approach which also captures alterations due to the epigenetic memory of the ESC-derived fibroblastoid cells used for the generation of isogenic iPSCs. Therefore, we became interested in the question which reprogramming-associated changes would occur in an ‘circular’ isogenic system where somatic cells are reprogrammed into iPSCs and subsequently re-differentiated into the original somatic phenotype—a setting which should largely exclude somatic memory.

We chose long-term self-renewing neural stem cells (NSCs) [15] as the somatic cell type for the study. This is a highly stable NSC population that has been broadly used in disease modeling and experimental cell transplantation [15,16]. Specifically, the female human ESC lines H9.2 and I3 were differentiated into this NSC type, reprogrammed into iPSCs, and subsequently re-differentiated into the very same NSC type. Global expression and DNA methylation profiling was performed for both NSC populations and their parental pluripotent cells of origin, revealing a high degree of similarity between isogenic ESC- and iPSC-derived NSCs at the autosomal level. In contrast, X chromosomal gene expression and DNA methylation differed markedly between parental and reprogrammed cells, indicating that even in a scenario where heterologous epigenetic memory is excluded by re-differentiating iPSCs back into their parental lineage, the X chromosome is particularly prone to reprogramming-associated alterations.

## 2. Results

### 2.1. Reprogramming and Subsequent Re-Differentiation of Human NSCs

**Figure 1:**
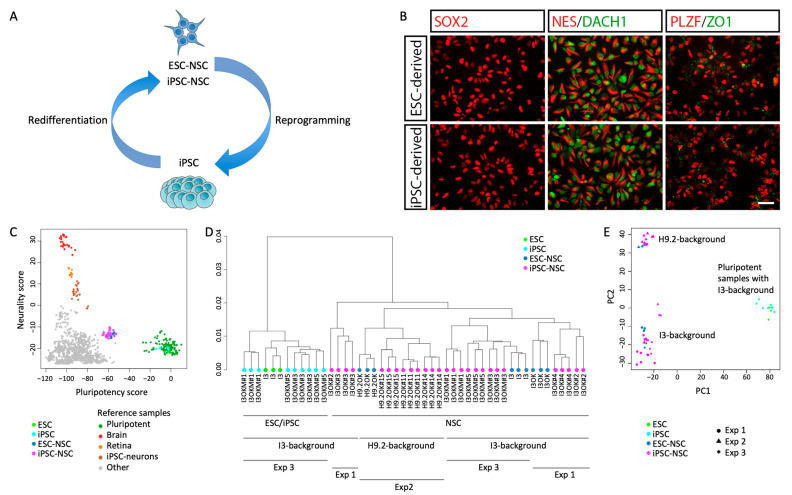
Derivation of isogenic iPSC-derived NSCs and their global transcriptional analysis (**A**) Scheme of the isogenic NSC system established in this study. ESC-derived NSCs (ESC-NSC) were reprogrammed into iPSCs, which were subsequently re-differentiated into iPSC-derived NSCs (iPSC-NSC). (**B**) Both ESC- and iPSC-derived NSCs show a characteristic polar, rosette-like organization and express the characteristic NSC markers SOX2, nestin, DACH1, and ZO1. Shown are exemplary data from ESC-NSC line NSC^I3^ and iPSC-NSC line NSC^I3OK#4^. Scale bar: 50 µm. (**C**) Neurality-pluripotency analysis of ESC, iPSC, and NSC lines. ESC and iPSC lines cluster with public domain control pluripotent cell lines, whereas NSC lines cluster diagonally between neural tissue/iPSC derived neurons and pluripotent cells. Other cell types, e.g., fibroblasts show no neurality or pluripotency. (**D**) Global hierarchical clustering of gene expression data. (**E**) Principal component analysis shows separate clustering for NSC samples of different genetic backgrounds.

For the generation of an isogenic stem cell system based on human ESC- and iPSC-derived NSCs we applied previously described long-term self-renewing neuroepithelial stem cells, also known as lt-NES cells [15] derived from the ESC lines I3 and H9.2 (Figure 1A). These cells can be continuously propagated in the presence of the growth factors FGF2 and EGF, exhibit a defined regional ventral hindbrain identity and develop into functional neurons following growth factor withdrawal [15,16]. They are characterized by their typical rosette-like growth pattern and homogeneously express the neural stem cell markers SOX2 and NESTIN, as well as the rosette-associated transcription factors DACH1 and PLZF (Figure 1B). Reprogramming of ESC-derived NSCs was performed by overexpression of OCT4 and KLF4 alone or in combination with cMYC. To that end we transduced the NSCs with either doxycycline-inducible lentiviral vectors, an approach similar to the generation of ‘secondary’ iPSC from iPSC-derived fibroblasts carrying inducible reprogramming factors [17], or with Sendai viral vectors (for an overview on the generated lines and applied reprogramming techniques see Supplementary Figure S1A). The resulting pluripotent lines were characterized for expression of the pluripotency-associated markers OCT4, TRA-1-60 and TRA-1-81 (Supplementary Figure S1B). Furthermore, their differentiation potential into cells of all 3 germ layers was confirmed by embryoid body formation and subsequent immunofluorescence analysis with antibodies to smooth muscle actin (SMA, mesoderm), alpha-fetoprotein (AFP, endoderm) and beta-III-tubulin (TUBB3, ectoderm; Supplementary Figure S1C). We then re-differentiated these iPSCs into NSCs (iPSC-NSCs) using the same protocol used to generate the donor NSCs from ESCs [15]. Resulting iPSC-NSCs exhibited a marker expression profile and differentiation potential comparable to ESC-derived NSCs (Figure 1B, Supplementary Figure S1D).

### 2.2. Maintenance of Autosomal and Alteration of X Chromosomal Gene Expression after Circular Reprogramming of Human NSCs

We next investigated gene expression profiles of iPSC-NSCs in comparison to ESC-NSCs. The complete panel included 9 iPSC-NSC lines and their matched ESC-derived counterparts (three ESC-NSC lines) generated in 3 independent experiments and hybridized in biological triplicates (Supplementary Figure S1A). As reference, one parental ESC line (I3; triplicates) and three iPSC lines derived thereof (each in triplicates) were included. Comparing the transcriptional data with a reference dataset consisting of 98 pluripotent and 1028 non-pluripotent samples [18], PluriTest analysis showed clustering of the NSC lines in close vicinity on the path from pluripotent to neuronal cells (pluripotency score vs. neurality score) independent of their cellular origin (Figure 1C; Supplementary Figure S2A). Hierarchical clustering showed (i) separation of pluripotent and neural stem cells, (ii) separation of neural stem cells with respect to their genetic background and (iii) separation of the cell lines with respect to the individual experiment but no clear separation of ESC-NSCs and iPSC-NSCs in the context of all experiments (Figure 1D). Principal component analysis confirmed the separation of pluripotent cells and neural stem cells (PC1) and the clustering of neural stem cells with regard to their genetic background, which was independent of whether they are ESC- or iPSC-derived (Figure 1E).

**Figure 2:**
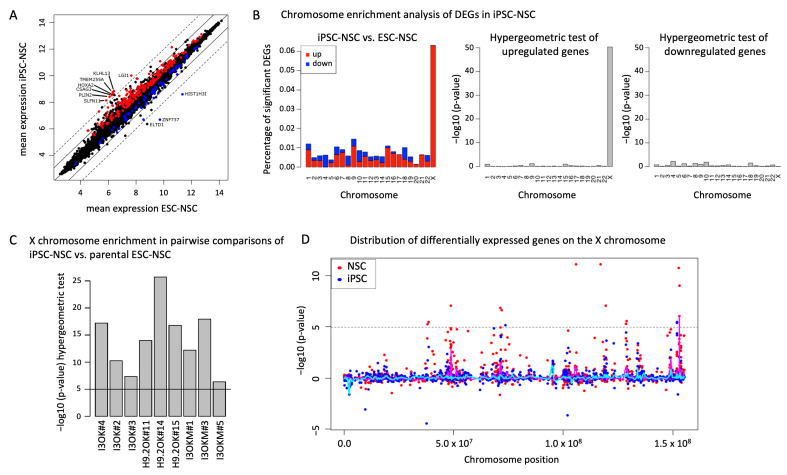
Differential expression is enriched on the X chromosome. (**A**) Scatter plot of global gene expression patterns comparing iPSC-derived NSC with ESC-derived NSC. Dotted and solid lines indicate fold change of >4 and >2 in gene expression levels, respectively. Upregulated and downregulated fractions are indicated in red and blue, respectively. (**B**) Chromosome enrichment analysis of differentially expressed genes (DEGs) in a comparison of iPSC- and ESC-derived NSCs. Graphical representation of significant genes (adjusted *p*-value < 0.01) in percentages of measured genes on a given chromosome. Upregulated and downregulated fractions are depicted in red and blue, respectively and in hypergeometric tests of upregulated and downregulated genes. (**C**) Pairwise comparisons of iPSC-NSC lines with their corresponding parental ESC-derived NSC lines (NSC^I3OK#2^, NSC^I3OK#3^, NSC^I3OK#4^ vs. stable ESC-NSC line NSC^I3OK^; NSC^H9.2OK#11^, NSC^H9.2OK#14^, NSC^H9.2OK#15^ vs. stable ESC-NSC line NSC^H9.2OK^; NSC^I3OKM#1^, NSC^I3OKM#3^, NSC^I3OKM#5^ vs. ESC-NSC line NSC^I3^). X chromosome enrichment was significant for all pairwise comparisons (*p*-value < 10^−5^). *p*-value cutoff for individual genes: 0.05 (*t*-test, Benjamini–Hochberg-adjusted). (**D**) Distribution of differentially expressed genes across the X chromosome. Pink and cyan lines indicate local enrichment of differentially expressed genes in NSCs (red) and pluripotent cells (blue), respectively.

When comparing expression profiles of ESC-NSCs and iPSC-NSCs in more detail, we found a remarkable similarity between ESC- and iPSC-derived NSC lines with only minute differences (Pearson correlation coefficient: 0.98). Surrogate Variable Analysis (SVA) [19] with adjusted *p*-value < 0.01 (Benjamini–Hochberg) showed only 264 genes to be significantly differentially expressed, and only 36 of these genes deviated at a 2-fold difference (Figure 2A). Among these genes, no gene ontology was enriched in ESC- or iPSC-derived NSCs (DAVID; *p*-value < 0.05; [20]). However, gene set enrichment analysis (GSEA) [21,22] revealed that 13 of the 36 differentially expressed genes (adjusted *p*-value = 0.00004058; *LINC00461*, *KLHL13*, *CDYL2*, *ZDHHC15*, *LRCH2*, *SLFN11*, *EPB41L3*, *SLITRK3*, *CXORF57*, *ZNF215*, *KIF1A*, *ASTN1*, *RBM11*) are associated with the histone modifications gene set H3K27me3 in SK-N-SH cells (H3K27me3_SK-N-SH_hg19, containing more than 2200 genes). At the same time, we found that many of the differentially expressed genes, such as *TMEM255A*, *KLHL13*, *CSAG3*, or *ZDHHC15*, are located on the X chromosome.

We therefore investigated the genomic localization of differentially expressed genes (adjusted *p*-value < 0.01) and plotted their proportion against chromosome number (Figure 2B). Indeed, this analysis revealed a significant overrepresentation of differentially expressed genes on the X chromosome (72 out of 264 genes; 8 out of the 36 genes with a > 2-fold deviation; Supplementary Table S1). Interestingly, all of the differentially expressed genes on the X chromosome showed a higher expression in iPSC-NSCs compared to ESC-NSCs, whereas on autosomal chromosomes no such enrichment was detectable (Figure 2B). X chromosome enrichment of differentially expressed genes between ESC- and iPSC-derived NSCs was also significant for all individual pairwise comparisons (*p*-value < 10^−5^, *p*-value cutoff for individual genes: 0.05, *t*-test, Benjamini–Hochberg-adjusted; Figure 2C) regardless of their genetic background or the transgenes used for reprogramming.

It is well known that female human pluripotent stem cells can undergo an erosion of X chromosome inactivation (XCI) over time in culture, characterized by loss of *XIST* expression and foci of H3K27 trimethylation, as well as transcriptional de-repression of genes on the inactive X [23,24]. As both genetic backgrounds included in this study are of female origin and, as such, an erosion in the pluripotent state can be carried over to somatic cell types derived from them, we were interested in whether transcriptional alterations were already detectable at the iPSC state. To that end, we analyzed the expression pattern of three iPSC lines and their matched ESC counterpart. Indeed, chromosome enrichment analysis of differentially expressed genes also revealed a significant over-representation of X chromosomal genes in the analyzed iPSCs compared to their ESC counterpart (Supplementary Figure S2B). Furthermore, several of those differentially expressed X chromosomal genes showed a joint upregulation in iPSCs and iPSC-derived NSCs, whereas just a few autosomal genes showed a joint upregulation or downregulation in pluripotent and neural cells. This might indicate that differential expression of these X chromosomal genes was already present in the pluripotent iPSC state and subsequently maintained during differentiation into NSCs (Supplementary Figure S2C,D).

To clarify, we analyzed the transcriptional data of ESC- and iPSC-NSCs separately for autosomal genes and X chromosomal genes. Irrespective of whether all genes, autosomal genes or X chromosomal genes were analyzed, a separation according to the genetic background was observed in principle component 1 (PC1; Supplementary Figure S3A–C, red circles). Interestingly, no clear separation of ESC-NSCs and iPSC-NSCs was detected in principle component 1 or 2 when analyzing all genes or autosomal genes only, whereas when investigating X chromosomal genes only, a separation of ESC- and iPSC-derived cells became evident (blue circles). This is also reflected in the Pearson correlation coefficient, with about 0.98 and 0.97, in autosomal genes and X chromosomal genes respectively. Furthermore, the distribution of differentially expressed genes on the X chromosome reveals a regional accumulation in p11.23, q13.1, q22.3, q25/q26.1, and q28, which may indicate a partial X chromosome reactivation in these regions (Figure 2D). Together, these data show that isogenic human NSC lines propagated through a stable iPSC state maintain a remarkably similar autosomal gene expression profile and that many of the transcriptional changes observed for X chromosomal genes are already present in the pluripotent iPSC state.

### 2.3. Alterations in X Chromosomal DNA Methylation during NSC Propagation through a Stable iPSC State

To analyze whether the transcriptional changes might be associated with alterations in DNA methylation, DNA from three I3-iPSC-derived NSC lines and their matched ESC-derived counterpart (all in biological duplicates) were bisulfide converted and hybridized to an Illumina Infinium Human Methylation 450K bead chip. We observed a highly similar methylome of ESC- and iPSC-derived NSCs with a correlation of about 96.5% (Supplementary Figure S4A,B).

On the gene level, the most prominent alteration in methylation occurred within the genes *VENTX* and *PNPLA4*. However, hypomethylation of *VENTX* did not result in an altered expression in none of the analyzed iPSC-derived lines, and hypermethylation of the X chromosomal gene *PNPLA4* was associated with a significant reduced expression (*p*-value < 0.02) exclusively in the corresponding three analyzed I3-iPSC-derived NSC lines but not in any other line. Moreover, an enrichment of imprinted genes could be detected amongst all genes with at least one differentially methylated CpG (delta-beta > 0.2, *p*-value = 0.01426, www.geneimprint.com (accessed on 7 February 2024)).

**Figure 3:**
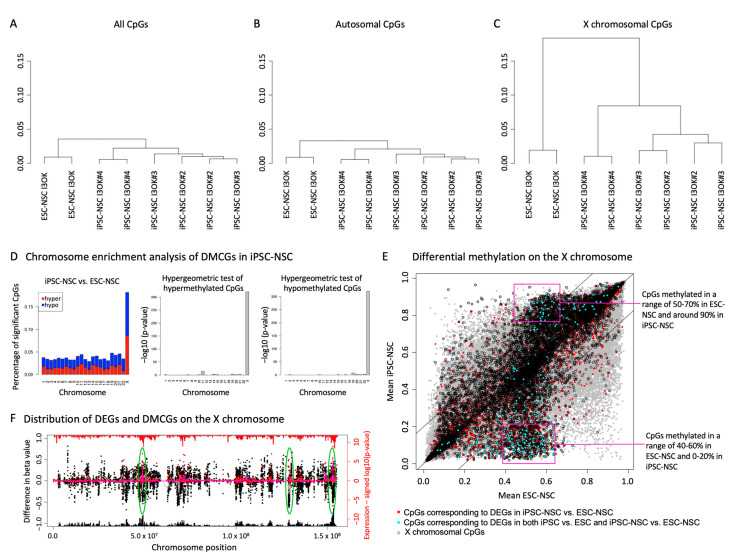
Enriched differential DNA methylation on the X chromosome coincides with differential gene expression. Global hierarchical clustering of DNA methylation data (**A**) of all analyzed CpGs, (**B**) autosomal CpGs, and (**C**) X chromosomal CpGs. (**D**) Chromosome-enrichment analysis of differentially methylated CpGs (DMCGs) in iPSC-NSC vs. ESC-NSC (delta beta > 0.2), depicted as the percentage of differentially methylated CpGs related to all analyzed CpGs on the respective chromosome. Hyper- and hypo-methylated fractions are depicted in red and blue, respectively and in hypergeometric tests of hyper- and hypo-methylated CpGs. (**E**) Scatter plot of global DNA methylation patterns comparing iPSC-NSC and ESC-NSC. Lines indicate a difference in beta > 0.2. (**F**) Distribution of differentially expressed and methylated sites across the X chromosome are indicated with red and black dots, respectively. All analyzed probes and CpGs are indicated with red and black histograms on the outer axis, respectively. Pink and gray lines indicate local enrichment of differentially expressed genes and methylated CpGs comparing iPSC-NSC with ESC-NSC, respectively. Green circles indicate coherent differences in gene expression and DNA methylation.

Similar to what we had observed when analyzing gene expression profiles, hierarchical clustering revealed a much more profound separation of ESC- and iPSC-derived NSCs when investigating X chromosomal CpGs compared to the analysis of all CpGs or autosomal CpGs only with a correlation of 85% (X chromosomal CpGs) and 96.5% (autosomal CpGs genes), respectively (Figure 3A–C). Furthermore, plotting differentially methylated CpGs by chromosome revealed a clear overrepresentation of differentially methylated CpGs on the X chromosome (delta-beta > 0.2; Figure 3D). Interestingly, many X chromosomal CpGs were either hypermethylated or hypomethylated in iPSC-derived NSCs, whereas the ESC-derived NSCs showed in those CpGs a beta-value of approximately 0.5 (Figure 3D,E). Moreover, local enrichment of differentially methylated CpGs in distinct X chromosomal regions partially overlapped with the local enrichment of differentially expressed X chromosomal genes, indicating an association of altered X chromosome methylation and gene expression (Figure 3F, compare Figure 2D).

In summary, ESC- and iPSC-derived NSCs showed very similar autosomal methylation patterns, whereas X chromosomal methylation was altered with differentially methylated regions partially corresponding to sites exhibiting altered gene expression levels.

### 2.4. Biallelic X Chromosomal Gene Expression and X Chromosome Inactivation Status

We wondered whether the differences in gene expression and DNA methylation patterns between reprogrammed and non-reprogrammed cells are based on alterations in the XCI status and thereby associated with a de-repression of formerly inactivated X chromosomal alleles or whether they merely represent differences in the expression level of activated alleles. Therefore, we analyzed *XIST* expression in ESC- and iPSC-derived NSCs as well as fetal hindbrain neuroepithelial stem cells, a primary cell population which underwent regular in vivo differentiation and should thus display regular XCI. Transcriptional analysis of *XIST* expression in NSCs revealed no significant differences before and after reprogramming, but lower expression levels in ESC- and iPSC-derived lines than in primary neural stem cells (Supplementary Figure S5A). This could point to an erosion of this inactivation mark already in the ESC-derived starting populations. Interestingly, the expression of XIST seems to be biallelic in the analyzed cell lines, while TSIX, its antisense RNA, appears to be monoallelically expressed (Supplementary Figure S5B,C).

XCI is further characterized by the formation of heterochromatin. Analysis of the XCI status via immunocytochemical staining for the repressive histone mark H3K27me3 revealed prominent large specks compatible with Barr body formation in primary NSCs, while such larger specks were missing in most of the pluripotent derivatives irrespective of ESC- or iPSC-origin, which also implies that deterioration of X inactivation had already started before the reprogramming process and not been reestablished thereafter (Supplementary Figure S5D).

To investigate whether reprogramming actually led to a biallelic expression of X chromosomal genes, we sequenced PCR-products of the X chromosomal genes *HEPH* and *TMEM255A*, which contain heterozygous SNPs. We identified a distinct loss of monoallelic expression of *HEPH* only in one iPSC-derived NSC line, whereas the X chromosomal gene *TMEM255A* remained monoallelically expressed (Supplementary Figure S6A). *HEPH*, which contains heterozygous SNPs in both genetic backgrounds (I3 and H9.2), showed differential expression only in a few iPSC-derived NSC lines, whereas *TMEM255A* was differentially expressed in all analyzed iPSC-derived lines when compared to ESC-derived NSCs (FC > 2; Supplementary Figure S6B). Interestingly, the iPSC-derived NSC line showing biallelic expression exhibited no increase in *HEPH* expression when compared to ESC-derived NSCs. Conversely, iPSC-derived NSCs exhibiting increased *HEPH* and *TMEM255A* expression levels showed no evidence of reprogramming-associated biallelic expression (Supplementary Figure S6B). Taken together, these two exemplary X chromosomal genes show little evidence of the erosion of monoallelic expression and no strict correlation of allelic expression pattern and transcript levels.

## 3. Discussion

Reprogramming to pluripotency is a complex process, with the requirement of massive epigenetic remodeling that might affect diverse cellular characteristics. Several studies have already addressed the question whether iPSCs and ESCs differ in their transcriptional profiles and revealed hundreds to thousands of differentially expressed genes [1,2]. However, the number of differentially expressed genes was much lower when comparing isogenic iPSC and ESC lines, to a range of 102–154 in female [25] and 0–49 in male iPSCs [10,13]. Recent data suggest that many of these alterations relate to epigenetic memory reflecting the source cells used for iPSC generation [14]. We reasoned that detection of mere reprogramming-associated changes without co-detection of somatic memory-related differences might be possible when a defined somatic cell population is propagated through an iPSC state and then re-differentiated into the same lineage.

To that end, we chose a human ES cell-derived NSC generated according to an established protocol that yields highly comparable NSC populations [15]. Data from this isogenic system indeed show a remarkably similar transcriptome for ESC- and iPSC-derived NSCs, with only minor transcriptional differences that were even smaller than differences due to different genetic backgrounds or inter-experimental variation. These findings are consistent with observations of Choi and colleagues in a male isogenic reprogramming system in which no consistent differences in gene expression signature could be detected, and transcriptional variation mainly correlated with the genetic background of the analyzed cells [13].

Our results are also in line with the recent study by Buckberry et al. (2023), who found that during reprogramming, transient propagation of the cells under conditions promoting a naïve pluripotent state transient can correct epigenetic and functional deficits occurring during classic reprogramming into a primed state and thus make the resulting human iPSCs more similar to human ESCs [14]. In the context of this study, which also emphasizes the impact of culture conditions on the quality of the pluripotent state, they compared ESCs and isogenic iPSCs generated from ESC-derived fibroblast-like cells and detected almost 1000 differentially expressed genes. Interestingly, these transcripts were enriched for mesoderm development, suggesting epigenetic memory to the parental fibroblast-like cells. The fact that our comparison of ESC-NSC and iPSC-NSC revealed only a small number of differentially expressed genes is in line with the notion that the reprogramming of a defined somatic cell population and its subsequent redifferentiation into the same somatic cell type annihilates differences due to somatic memory. Concurrently, the possibility that redifferentiation into the same lineage might itself be favored by epigenetic memory has not yet been taken into account. Another question evolving from this thought is whether such a facilitated entry into the neural lineage would also support redifferentiation into other neural cell types such as, e.g., neural crest stem cells.

Interestingly, and in contrast to the close similarities in the expression and methylation of autosomal genes and loci, we noted pronounced differential expression of X chromosomal genes, which coincides with X chromosomal regions exhibiting altered DNA methylation. These data are in agreement with findings from Teichroeb and colleagues. Similar to their observations, we could identify the X chromosomal regions p11.4 and q24 as hotspots of differentially expressed genes, but also the transcript TMEM255A [25]. However, we also detected local enrichment of hypermethylation on the X chromosome in iPSC-derived NSCs, although with no significant impact on overall transcription. It remains unclear whether this reflects further stabilization of an already non-transcribed status of X chromosomal genes or a general vulnerability of these regions to deviant methylation.

Our results are also consistent with previous findings showing that the majority of methylation differences between different female human pluripotent cell lines are located on the X chromosome [26]. The high similarity of autosomal methylation patterns, conversely, is in line with results from a previous study where we reported first data on this circular system with a focus on DNA methylation differences in isogenic ESC- and iPSC-derived neurons [27]. The X chromosomal changes uncovered in our current study might be related to partial erosion of XCI due to the reprogramming process and further propagation in the pluripotent state. It is known that, over time, in culture, pluripotent cells can undergo an erosion of XCI. Loss of both, *XIST* expression and foci of H3K27 trimethylation, as well as transcriptional de-repression of genes on the inactive X chromosome characterize an erosion of XCI, which cannot be reversed by either differentiation or further reprogramming [23]. However, we found evidence for monoallelic expression of the X chromosomal genes *TMEM255A* and *HEPH* before and after reprogramming, with the exception of biallelic expression of *HEPH* in one single iPSC-derived NSC line, which, however, did not result in elevated expression.

Interestingly, Vallot et al. found that X chromosomal gene reactivation upon XCI erosion occurs only in a subset of heterochromatin domains and genes [24]. However, they detected stable monoallelic expression of *HEPH* regardless of an eroded or inactive X chromosome status. This may indicate that the detected increase in X chromosomal gene expression after reprogramming may not be associated with a difference in XCI, but with a general susceptibility of X chromosomal gene expression to variation, and that biallelic expression of X chromosomal genes does not consistently lead to an increase in overall expression.

It is tempting to speculate that the X chromosomal DNA methylation, as one of the last remaining inactivation marks, is particularly susceptible to alterations during the reprogramming process due to its enormous epigenetic remodeling. This might also be true for histone modifications such as H3K27 trimethylation, as our identified differentially expressed genes were enriched for members of the H3K27me3-associated gene-set described for SK-N-SH cells. Interestingly, one of the most prominently upregulated genes in our iPSC-NSC, the E3 ubiquitin ligase adaptor protein KLHL13, was recently suggested to stabilize naïve pluripotency and delay differentiation kinetics in mouse ESCs [28]. Moreover, it was reported to be one of the late reactivated and upregulated genes during naïve mouse iPSC reprogramming [29,30]. However, its role in human pluripotency and differentiation remains yet to be defined.

In general, it would be interesting to explore whether naïve culture conditions for the pluripotent cells [31,32] or a transient naïve cultivation phase [14] can influence the susceptibility of the X chromosome to alterations in transcription and DNA methylation, as it was suggested to reverse erosion of XCI in pluripotent stem cells [33].

Several studies indicate possible consequences of reprogramming on the stability of genomic imprinting [25,34,35,36,37,38]. When comparing iPSC- and ESC-derived NSCs in our current study, a differential expression of one predicted imprinted gene (*ZNF215*) [39] could be detected, but no general bias towards imprinted genes on the expression level. Nonetheless, we noticed an enrichment of imprinted genes among all genes with at least one differentially methylated CpG, suggesting a susceptibility of reprogramming to perturb imprinted gene regulation.

Together, our data demonstrate a remarkable similarity in autosomal gene expression and DNA methylation patterns in human NSCs before and after propagation through an iPSC state. In contrast, overt differences are found for the X chromosome. Since our approach represents a circle from NSC to iPSC and back to NSC, these differences cannot be ascribed to somatic memory. Therefore, the X chromosomal alterations are most likely due to reprogramming-associated causes. However, while our data on XIST and H3K27me3 expression suggest that ESC-derived NSCs exhibit a significant degree of erosion of XCI already before reprogramming, it is conceivable that continued erosion of XCI contributes to the pronounced X-associated epigenetic differences observed upon reprogramming.

Naturally, several important points remain to be addressed in further studies. This relates in particular to the functional implications of the observed reprogramming-associated alterations in neural derivatives. Another question is whether the application of such a ‘circular’ system to other stably proliferating somatic precursors and different developmental stages results in similar alterations. From a methodological point of view, single cell analyses rather than bulk samples are likely to provide more details with respect to, e.g., changes in cellular heterogeneity or selective accumulation of epigenetic alterations in distinct subpopulations.

The reprogramming of defined somatic populations with subsequent re-differentiation of the resulting iPSCs into the same somatic cell type may, in general, represent a useful approach to study reprogramming-associated alterations in gene transcription and DNA methylation without confounding changes due to somatic memory. In conjunction with results of several previous studies, our findings support the notion that the X chromosome is particularly prone to reprogramming-associated alterations impacting gene expression and DNA methylation. Such changes deserve particular attention when using female iPSC-derived cells for disease modeling and regenerative applications, as differences in X chromosomal gene expression may directly or indirectly confound data interpretation.

## 4. Materials and Methods

### 4.1. Cell Culture

Human ESCs (I3, https://www.wicell.org/home/stem-cells/catalog-of-stem-cell-lines/te03.cmsx?closable=true (accessed on 7 February 2024); H9.2, https://hpscreg.eu/cell-line/WAe009-A-2 (accessed on 7 February 2024)) and iPSCs were cultured on mouse embryonic fibroblasts (MEFs) according to standard protocols, or under feeder-free conditions on Matrigel (BD Biosciences Franklin Lakes, NJ, USA) in E8 medium. ESC-derived NSCs (H9.2 and I3) were generated and maintained on polyornithine/laminin (Merck, Darmstadt, Germany; Thermo Fisher Scientific, Waltham, MA, USA) with EGF and FGF2 (BD Biosciences) as described [15]. iPSC-derived NSCs were derived and cultured according to the same protocol. Default differentiation for NSC quality control was performed on Matrigel (BD Biosciences) in differentiation medium devoid of growth factors [15].

### 4.2. Reprogramming of NSCs to Pluripotency

For reprogramming via the lentiviral TetON-system, stable inducible NSC lines harboring the reprogramming factors OCT4 and KLF4 (pLVXTP-Tet-On (Takara Bio, Kusatsu, Japan); FUW-OCT4 and FUW-KLF4 (Addgene, Watertown, MA, USA)) were induced to reprogram by the addition of 1 µg/mL doxycycline (Merck) to the culture medium. After 24 h, cells were transferred to MEFs. Medium was switched to KOSR-medium supplemented with doxycycline and changed every other day. Doxycycline was withdrawn upon colony formation and doxycycline independent colonies were mechanically isolated and expanded. In case of integration free reprogramming with Sendai-virus, NSCs were infected with equal amounts of Sendai-viral particles of OCT4, KLF4 and cMYC by spinfection at 1500× *g*, 30 min and incubation for 8–14 h. After 24 h, cells were transferred to MEFs. The medium was switched to KOSR-medium and changed every other day. Colonies were mechanically isolated and expanded to establish iPSC lines.

### 4.3. Lentiviral Transduction of NSCs

The production of lentiviral particles was performed in HEK293FT cells (Thermo Fisher Scientific) by calcium phosphate transfection of expression constructs and helper plasmids, as previously described [40]. Viral particles were concentrated by ultracentrifugation at 19,600 rpm at 4 °C for 1.5 h in a Sorvall Surespin 630 rotor (Thermo Fisher Scientific) and resuspended in HBSS (Thermo Fisher Scientific). NSCs were exposed to virus containing media supplemented with 5 µg/mL polybrene (Merck) overnight at 37 °C and 5% CO_2_. NSCs were treated with puromycin (5 µg/mL, PAA) for at least 2 days to obtain stable cell lines harboring the reprogramming factors.

### 4.4. In Vitro Differentiation of Pluripotent Cells into Three Germ Layers

For undirected differentiation, embryoid bodies (EBs) were generated. First, pluripotent colonies were detached from MEF-coated plates with collagenase. In order to remove any remaining fibroblasts, floating aggregates were washed three times. EBs were allowed to sink in a 15 mL polypropylene tube and washing media was subsequently removed. To avoid attachment to the culture dish, aggregates were cultivated in non-adherent plates and EB-medium was changed every other day by sedimentation. EBs were plated on gelatin-coated dishes after propagation for at least 4 days in EB medium. Aggregates were allowed to adhere, and cultivation media was changed to MEF medium. Following 10 days of differentiation, outgrowth of diverse cell types could be detected.

### 4.5. Immunocytochemistry

Cells and EBs were fixed with 4% paraformaldehyde in PBS (supplemented with 1:500 glutaraldehyde for GABA staining) for 15 min at room temperature. Cells were washed with PBS and blocked for 30 min at room temperature with PBS containing 10% fetal calf serum for detection of TRA-1-60 and TRA-1-81 or with PBS containing 10% fetal calf serum and 0.1% Triton X-100 (Merck) for all other primary antibodies. Cells were incubated with primary antibody for 16 h at 4 °C, washed three times in PBS, incubated with secondary antibody for 1 h, counterstained with DAPI (1:10,000, Merck) and mounted in Mowiol 4–88 (Carl Roth, Karlsruhe, Germany). The following antibodies and concentrations were applied: SOX2 (1:500, R&D Systems, Minneapolis, MN, USA), NES (1:300, R&D Systems), DACH1 (1:50, Proteintech, Rosemont, IL, USA), PLZF (1:50, CalbiochemMerck), ZO1 (1:100, Thermo Fisher Scientific), OCT4 (1:400, Santa Cruz, Dallas, TX, USA), TRA-1-60 (1:500, Thermo Fisher Scientific), TRA-1-81 (1:500, Thermo Fisher Scientific), AFP (1:100, Agilent, Santa Clara, CA, USA), SMA (1:100, Agilent), TUBB3 (1:1500, Convance, Princeton, NJ, USA), MAP2ab (1:250, Merck), GABA (1:600, Sigma), GFAP (1:250, Agilent), H3K27me3 (1:1000, Merck Millipore, Burlington, MA, USA), Alexa488 anti-rabbit IgG (1:1000, Thermo Fisher Scientific), Alexa555 anti-mouse IgG (1:1000, Thermo Fisher Scientific), and Alexa555 anti-mouse IgM (1:1000, Thermo Fisher Scientific).

### 4.6. Transcriptome Analysis

Isolation of total RNA was carried out with RNeasy Mini Kit (Qiagen, Hilden, Germany) with on column DNase digestion following the manufacturer’s instructions. The integrity of isolated total RNA was examined using BioAnalyzer 2100 (Agilent), following the instructions of the manufacturer’s protocol. All RNA samples used in this study showed intact 28S and 18S ribosomal RNA signals and RNA integrity number (RIN) > 9.5. Whole transcription profiles were generated using HumanGene1.0stv1 microarrays (Thermo Fisher Scientific), according to the manufacturer’s instructions. Data was preprocessed with the Robust Multichip Average (RMA) method using apt-probeset-summarize from the Affymetrix Power tools software suite (Version 2.11.6) and further analyzed using the Multi Experiment Viewer (MeV, part of TM4 Microarray Software Suite (https://www.sciencedirect.com/science/article/pii/S0076687906110095 (accessed on 7 February 2024)) and R (Version 3.1.2). Hierarchical clustering was performed with Pearson correlation and average-linkage. For evaluation of pluripotency based on gene expression profiles, PluriTest was applied with a reference dataset consisting of 98 pluripotent and 1028 non-pluripotent samples [18]. We used the same reference dataset to construct a “neurality score”, using a similar approach as was used in PluriTest to determine the pluripotency score [41]. Briefly, we first performed non-negative matrix factorization with 8 components on the complete dataset. Afterwards, the eight components were ranked according to their area under the receiver operating characteristic for classification of cells into brain tissues (34 samples) and non-brain tissues (without retina and iPSC-derived neurons). Finally, a logistic regression model was calculated (allowing only for positive coefficients) that maximizes the margin between brain samples and non-brain samples [41]. Differentially expressed genes were identified via ‘Surrogate Variable Analysis (SVA)’ [19] to account for potential confounder effects, like batch effects (Benjamini–Hochberg adjusted *p*-value < 0.01). Chromosome enrichment analysis was performed using a hypergeometric test on the differentially expressed probes (using only the *p*-value criterion to define significant probes).

### 4.7. Whole Genome Methylation Analysis

Purification of genomic DNA was carried out with DNeasy Blood & Tissue Kit (Qiagen) following the manufacturer’s instructions. Genome-wide DNA methylation profiles were generated at the Institute for Human Genetics, Bonn University and at the Institute for Genetics and Epigenetics, Saarland University using Infinium HumanMethylation 450 K beadchip assay (Illumina, San Diego, CA, USA). Infinium methylation assay was performed according to the manufacturer’s instructions and fluorescently stained chips were imaged with iScan (Illumina). All methylation profiles were extracted and average normalized using GenomeStudio software (https://support.illumina.com/array/array_software/genomestudio/downloads.html (accessed on 7 February 2024)). CpGs with a difference in mean beta value greater than 0.2 were considered significant. Hierarchical clustering was performed with Pearson correlation and average linkage. Chromosome enrichment analysis was performed using a hypergeometric test on the differentially methylated CpGs.

### 4.8. SYBR Green-Based Quantitative Real-Time RT-PCR Analyses

cDNA was synthesized using the iScript Reverse Transcription (RT) Kit (Qiagen), following the manufacturer’s instructions. Quantitative real-time RT-PCR was performed with SYBR Green PCR Kit (Qiagen) and an Eppendorf Mastercycler. Data were normalized to 18S rRNA levels. PCR products were assessed by dissociation curve and gel electrophoresis. The following primers were used: XIST, forward, TTGGATTTGGCCTGCTGTTC; reverse, CAGGGACAGGCACAGAAAAG; 18S rRNA, forward, ATTCTTGGACCGGCGCAA; reverse, CCGACCGGCGATGCGGC.

### 4.9. Allele Specific Expression Analysis

PCR products were purified via gel electrophoresis and subjected to Sanger sequencing. Sequencing traces were analyzed using Ape. Genomic DNA was used to identify the presence of heterozygous SNPs. See Supplementary Table S2 for primer sequences and SNP information.

### 4.10. Statistical Analysis

The data are presented as mean + SEM. Statistical significance, unless otherwise stated, was analyzed by two-tailed Student’s *t*-test for control and experimental conditions, and *p* < 0.05 was considered to be statistically significant.

## Data Availability

Gene expression and DNA methylation data are available from the authors upon request.

## Supplementary Materials

The following supporting information can be downloaded at: https://www.mdpi.com/article/10.3390/ijms25063214/s1.
