# Supplementary material for: Epigenetic and Transcriptional Shifts in Human Neural Stem Cells after Reprogramming into Induced Pluripotent Stem Cells and Subsequent Redifferentiation
Source: Int J Mol Sci. 2024 Mar 12;25(6):3214. doi: 10.3390/ijms25063214 (PMC10969834; doi:10.3390/ijms25063214)
Supplement: Supplementary file 1 [file ijms-25-03214-s001.zip › ijms-2889085-supplementary.pdf]

S1

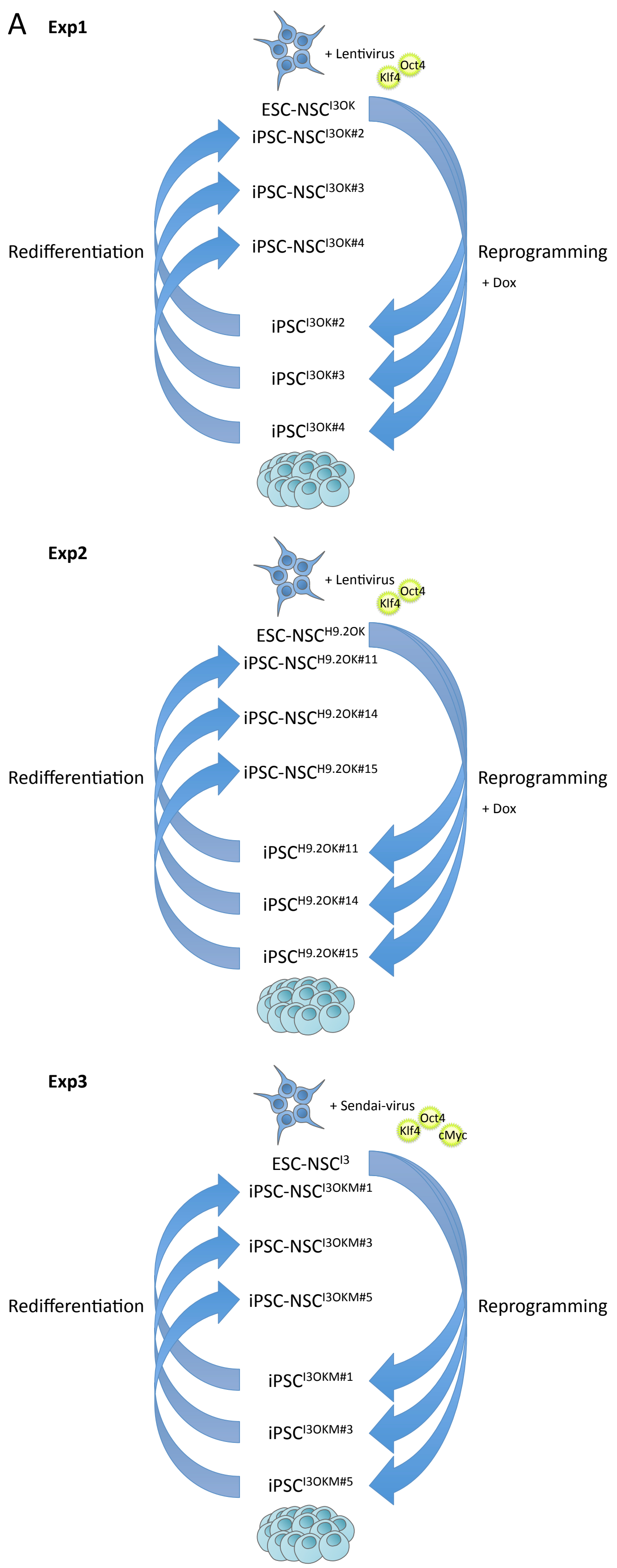

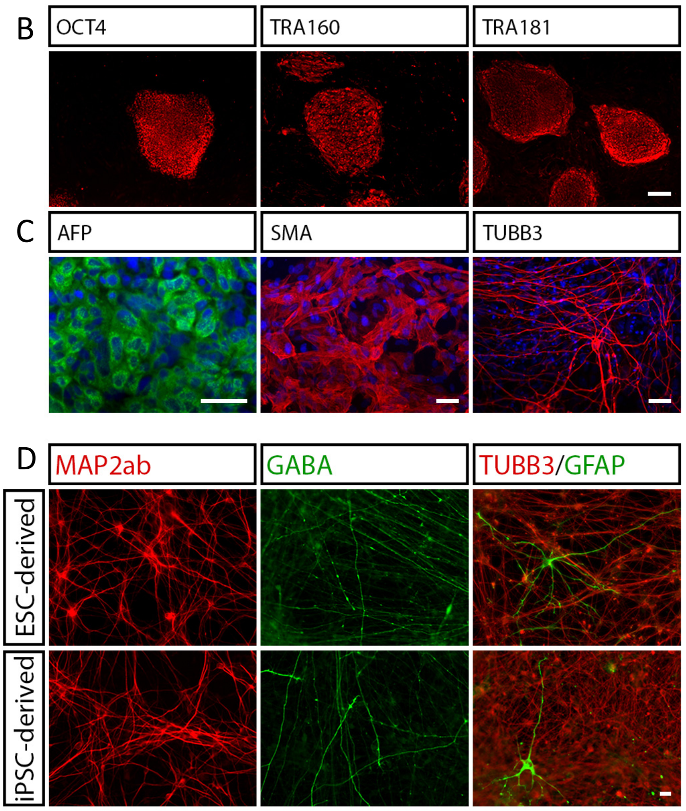

Figure S1. (A) Detailed schematic of cell line generation in the 3 experimental streams of the study. **(B)** Representative characterization of iPSC lines for pluripotency-related marker expression (data shown for iPSC$^{I3OK\#3}$; scale bar: 200µm) and **(C)** their differentiation potential into all three germ layers *in vitro*, indicated by expression of AFP (endoderm), SMA (mesoderm) and TUBB3 (ectoderm). Scale bar: 50µm. **(D)** iPSC-derived NSCs show comparable neural differentiation potential to ESC-derived NSCs with expression of the neuronal markers MAP2, TUBB3, the neurotransmitter GABA and the astrocytic marker GFAP (data shown: ESC-NSC^{I3}, iPSC-NSC$^{I3OK\#2}$; scale bar: 20µm).

S2

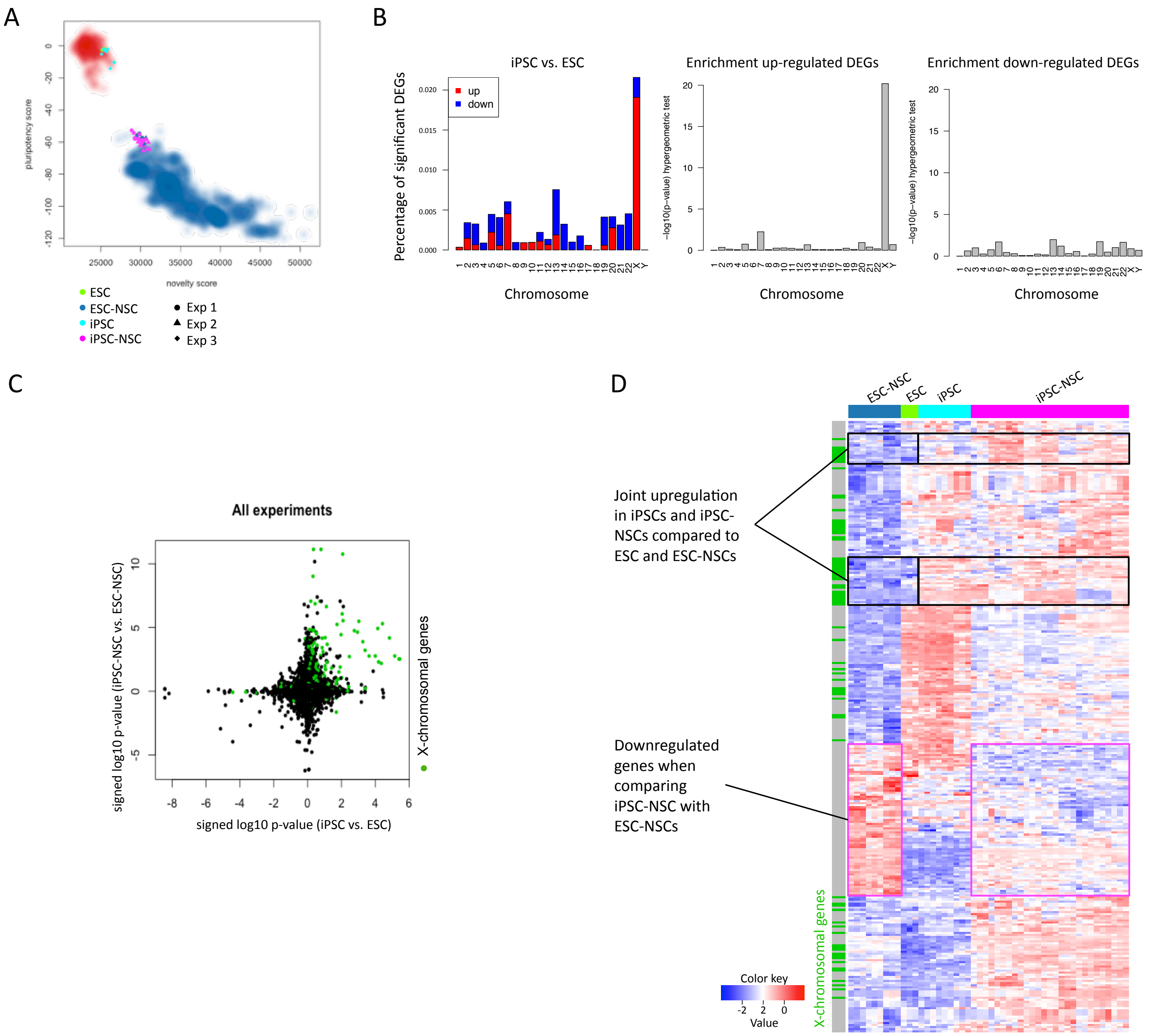

Figure S2. (A) PluriTest analysis of the transcriptional data from ESC and iPSC lines and their NSC derivatives shows proper reprogramming and redifferentiation. **(B)** Chromosome-enrichment analysis of differentially expressed genes (adjusted p-value <0.01). Graphical representation of the number of genes with fold change >2 differences when comparing iPSC with ESC in percentages of measured genes on a given chromosome. Upregulated and downregulated fractions are indicated in red and blue, respectively. Strong enrichment of up-regulated DEGs is further supported by the hypergeometric tests of up-regulated and down-regulated genes. **(C)** Scatter plot of global gene expression patterns comparing iPSC-NSCs with ESC-NSCs and iPSCs with ESCs. **(D)** Transcriptome heatmap of differentially expressed genes (p-value <0.01) comparing iPSC-NSCs with ESC-NSCs. X chromosomal genes are depicted in green.

S3

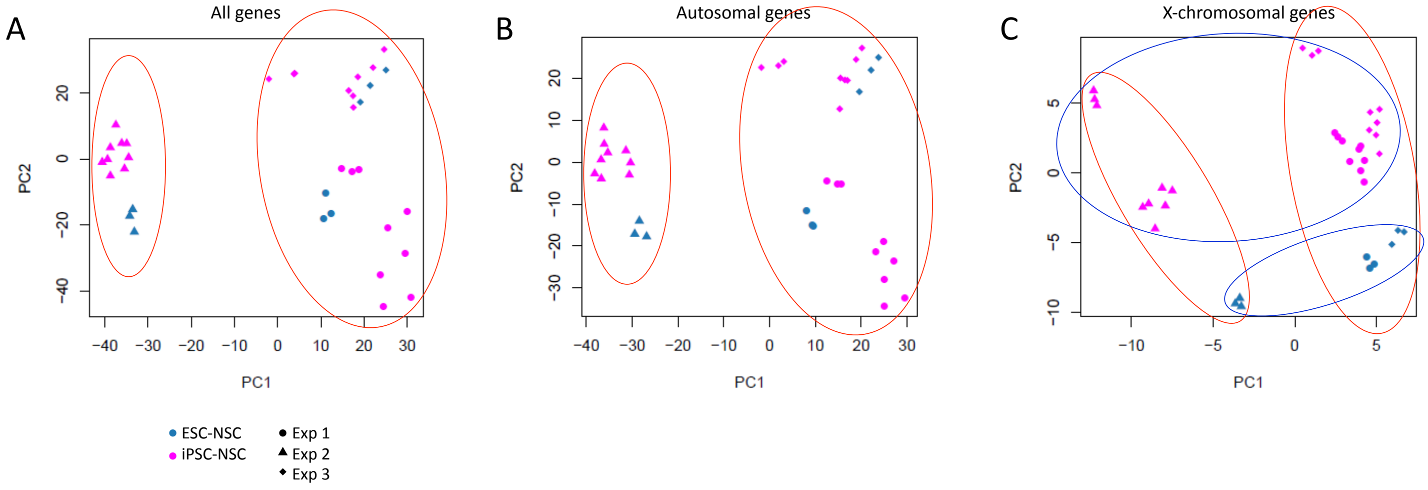

Figure S3. Principal component analysis of gene expression data (NSC samples only) of all analyzed genes **(A)**, autosomal genes **(B)** and X chromosomal genes **(C)**. PC2 shows a clear separate clustering of reprogrammed and non-reprogrammed NSC samples when analyzing X chromosomal genes only. Red and blue circles indicate separate clusters of different genetic backgrounds (I3 vs. H9.2) and ESC- and iPSC-derived samples, respectively.

S4

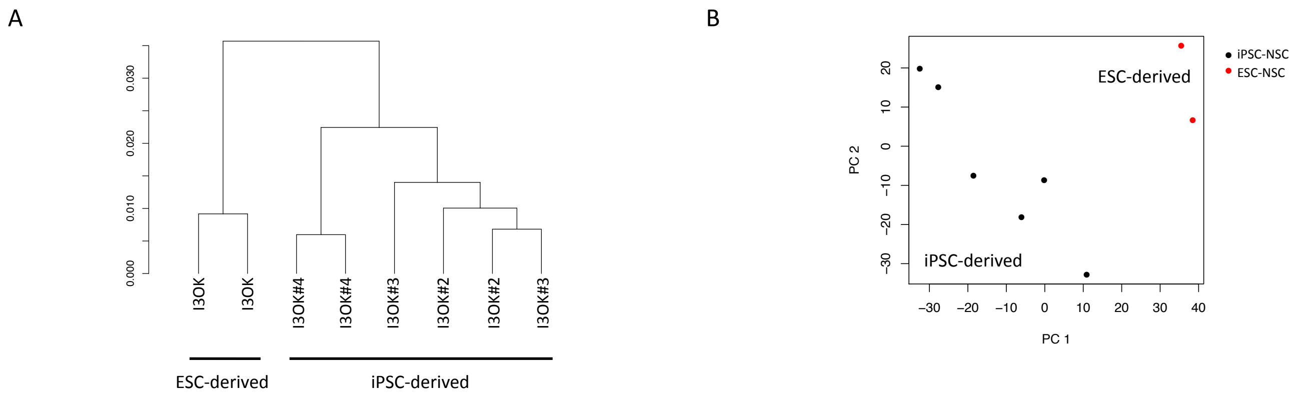

Figure S4. (A) Hierarchical clustering and **(B)** principal component analysis of global DNA methylation profiles of ESC- and iPSC-derived NSCs. DNA from three I3-iPSC-derived NSC lines and their matched ESC-derived counterpart (all in biological duplicates)

S5

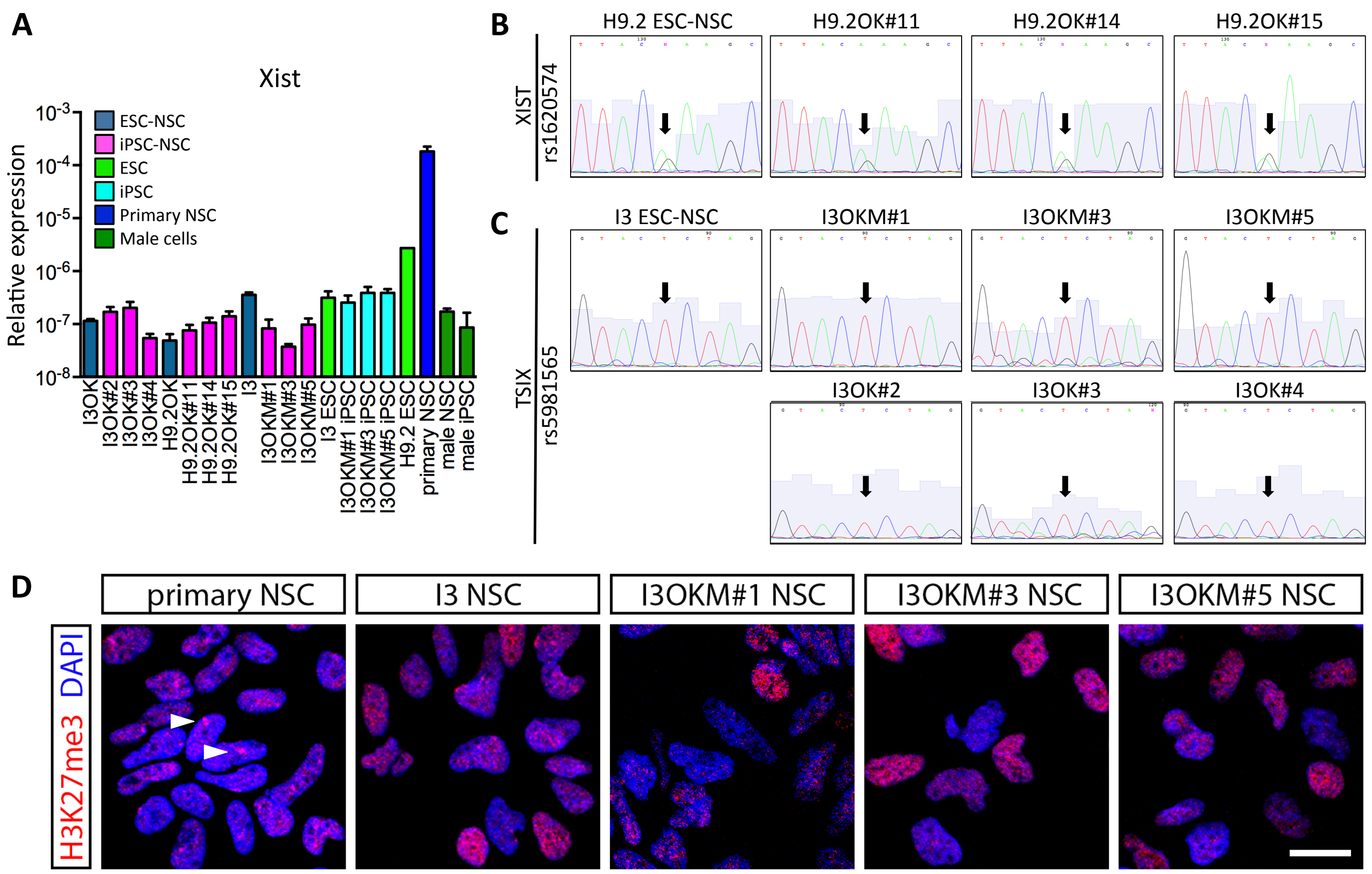

Figure S5. XCI status in iPSC-derived NSCs. **(A)** Relative XIST expression of ESC- and iPSC-derived NSCs. Primary NSC: female primary hindbrain neuroepithelial stem cell line SAi5 (Tailor et al., 2013); male NSC: I6 ESC-derived NSCs (https://hpscreg.eu/cell-line/TECHe003-A); male iPSC: I6 NSC-derived iPSCs. **(B, C)** Sequencing data of X chromosomal genes with heterozygous SNPs. iPSC-derived NSCs show a biallelic expression of XIST and a monoallelic expression of TSIX with no skewing to the other X chromosome after reprogramming. Arrows indicate the site of the heterozygous SNP. Blue background indicates Phred score for each base call. **(D)** H3K27me3 heterochromatin staining of ESC- and iPSC-derived NSCs. Arrow heads indicating H3K27me3 accumulation. Scale bar: 20µm.

S6

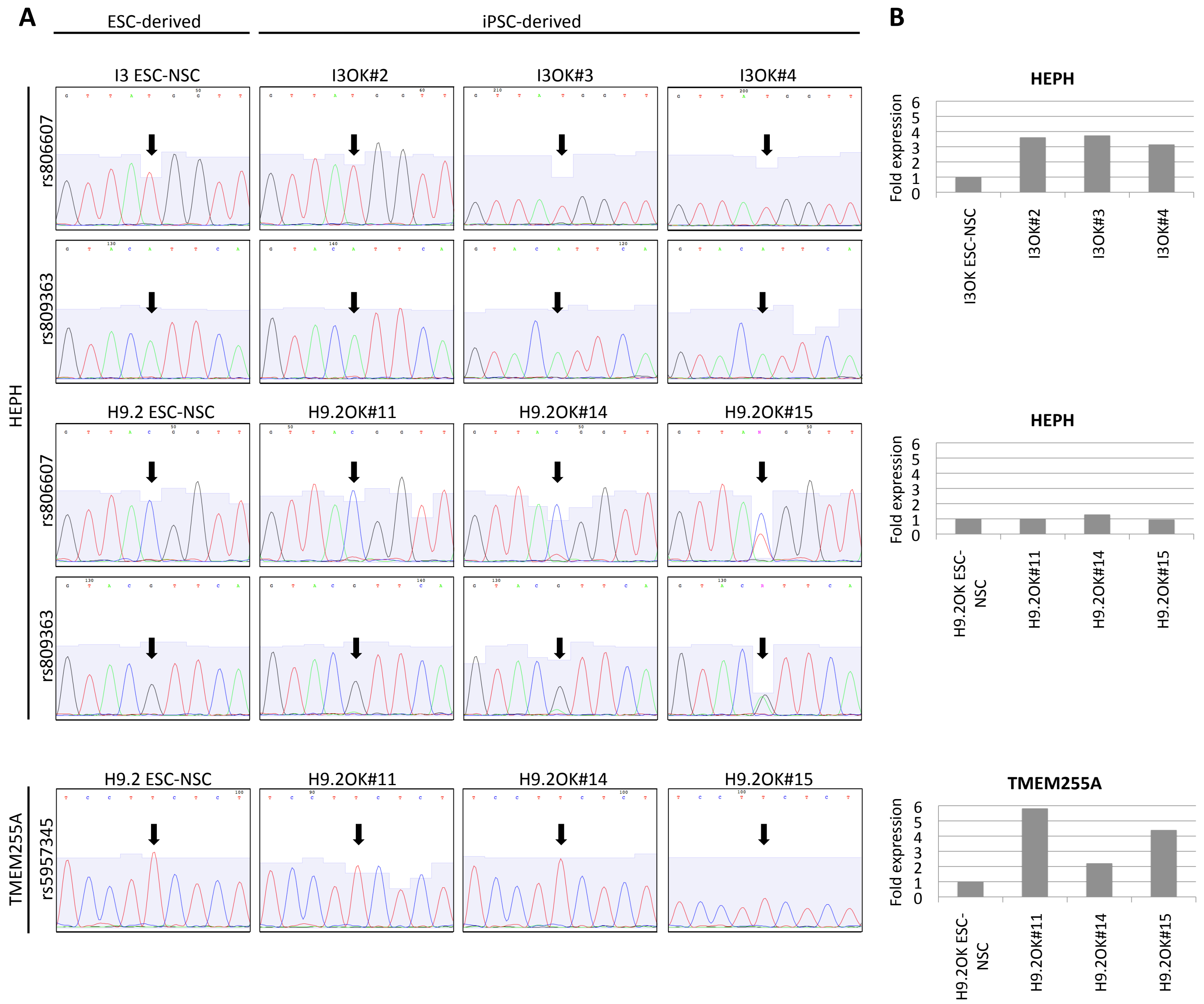

Figure S6. Allele-specific X chromosomal gene expression in iPSC-derived NSCs. **(A)** Sequencing data of X chromosomal genes with heterozygous SNPs. iPSC-derived NSCs show partial loss of monoallelic expression of the X chromosomal gene *HEPH* (rs806607, rs809363), whereas the X chromosomal gene *TMEM255A* (rs5957345) remains monoallelically expressed as confirmed by Sanger sequencing. Arrows indicate the site of the heterozygous SNP. Blue background indicates Phred score for each base call. **(B)** Corresponding fold expression of *HEPH* and *TMEM255A* in iPSC-NSCs.

Table S1

Table S1: List of differentially expressed genes in iPSC-NSCs vs. ESC-NSCs
(log2-fold change>1, adjusted p-value <0.01)

Differentially expressed genes in iPSC-NSCs vs. ESC-NSCs			
**Gene**	**Log-Fold change**	**Adj. p-value**	**Chromosome**
ZNF737	-3,04	1,01E-04	19
HIST1H3I	-2,76	1,13E-03	6
PECAM1	-1,85	6,26E-03	17
LRCH2	1,00	1,25E-04	X
SLC9A7	1,02	2,07E-04	X
EPB41L3	1,03	5,70E-04	18
CDYL2	1,04	6,75E-11	16
STPG1	1,08	3,34E-03	1
C21orf90	1,11	1,37E-04	21
ASTN1	1,15	2,80E-03	1
ANO5	1,16	7,48E-03	11
SLITRK3	1,18	6,01E-05	3
TGFB2	1,19	6,91E-05	1
KIF1A	1,20	6,91E-05	2
CXorf57	1,21	7,39E-12	X
STX3	1,26	3,07E-04	11
SOHLH2	1,26	5,20E-03	13
MAGEA2B	1,27	5,04E-03	X
RBM11	1,31	6,98E-03	21
ZNF215	1,31	4,23E-04	11
SYT11	1,34	9,85E-06	1
LINC00461	1,35	2,27E-03	5
TOM1L1	1,39	8,73E-03	17
SYT14	1,45	1,11E-03	1
COL1A2	1,56	6,35E-03	7
H1F0	1,56	4,04E-08	22
NFIA	1,62	7,52E-03	1
STMN2	1,67	3,77E-03	8
SLFN5	1,68	7,36E-07	17
ZDHHC15	1,98	1,08E-04	X
SLFN11	2,27	1,74E-03	17
PLIN2	2,31	2,38E-05	9
CSAG3	2,32	2,98E-03	X
LGI1	2,34	5,04E-03	10
KLHL13	2,42	7,39E-12	X
TMEM255A	2,44	8,66E-08	X

Table S2

Table S2: Primers for allele-specific expression analysis

Gene	SNP		Primer
HEPH	Rs806607	T/C	Fw: ACCAAGCTGCAAGAATCTACT<br>Rv: CCAGTGGCCAGACAGTAGTA
	Rs809363	A/G	
TMEM255A	Rs5957345	T/C	Fw: GCCCTATCTTTCCCTACCCC<br>Rv: ACATCCTGTTCCCCACTACTG
Xist	Rs1620574	A/G	Fw: GTGTCTTGGGTAGCAGAAGAAAA<br>Rv: CCGAGCCCCACAGAAAGTAA
Tsix	Rs5981565	C/T	Fw: GCTGGTGGGAGAAAGTATGG<br>Rv: AAGGCAAGATCAGCTAATACCA
